# 2-Carboxyl­atopyridinium–4-nitro­phenol (1/1)

**DOI:** 10.1107/S1600536814005650

**Published:** 2014-03-15

**Authors:** A. Sankar, S. Ambalatharasu, G. Peramaiyan, G. Chakkaravarthi, R. Kanagadurai

**Affiliations:** a Department of Physics, Presidency College, Chennai 600 005, India; b Department of Physics, CPCL Polytechnic College, Chennai 600 068, India

## Abstract

In the title 1:1 adduct, C_6_H_5_NO_3_·C_6_H_5_NO_2_, both mol­ecules are almost planar (r.m.s. deviations for the non-H atoms = 0.027 and 0.023 Å for 4-nitro­phenol and 2-carboxyl­atopyridinium, respectively). The pyridine mol­ecule crystallizes as a zwitterion (nominal proton transfer from the carb­oxy­lic acid group to the N atom in the ring). In the crystal, inversion dimers of the zwitterions linked by pairs of N—H⋯O hydrogen bonds generate *R*_2_^2^(10) loops; two 4-nitro­phenol mol­ecules link to the dimer by O—H⋯O hydrogen bonds, generating a four-molecule aggregate. These are linked by C—H⋯O inter­actions, forming a three-dimensional network.

## Related literature   

For a related structure, see: Pandi *et al.* (2012 ▶).
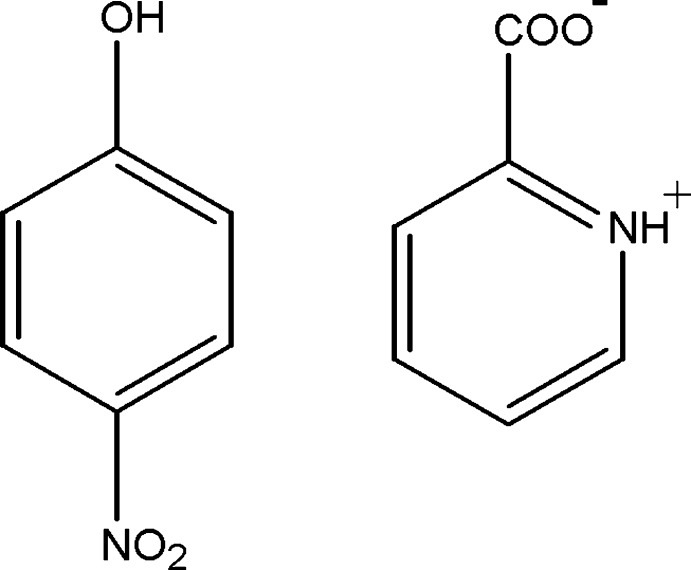


## Experimental   

### 

#### Crystal data  

C_6_H_5_NO_3_·C_6_H_5_NO_2_
*M* *_r_* = 262.22
Triclinic, 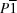

*a* = 6.1743 (4) Å
*b* = 7.0512 (3) Å
*c* = 14.2222 (8) Å
α = 101.727 (3)°
β = 92.191 (2)°
γ = 104.758 (4)°
*V* = 583.60 (6) Å^3^
*Z* = 2
Mo *K*α radiation
μ = 0.12 mm^−1^
*T* = 295 K
0.32 × 0.24 × 0.20 mm

#### Data collection  

Bruker Kappa APEXII CCD diffractometer
Absorption correction: multi-scan (*SADABS*; Bruker, 2004 ▶) *T*_min_ = 0.963, *T*_max_ = 0.977
13952 measured reflections
3486 independent reflections
2327 reflections with *I* > 2σ(*I*)
*R*_int_ = 0.028

#### Refinement  

*R*[*F* ^2^ > 2σ(*F* ^2^)] = 0.045
*wR*(*F* ^2^) = 0.130
*S* = 1.04
3486 reflections
180 parameters
2 restraints
H atoms treated by a mixture of independent and constrained refinement
Δρ_max_ = 0.14 e Å^−3^
Δρ_min_ = −0.24 e Å^−3^

### 

Data collection: *APEX2* (Bruker, 2004 ▶); cell refinement: *SAINT* (Bruker, 2004 ▶); data reduction: *SAINT*; program(s) used to solve structure: *SHELXS97* (Sheldrick, 2008 ▶); program(s) used to refine structure: *SHELXL97* (Sheldrick, 2008 ▶); molecular graphics: *PLATON* (Spek, 2009 ▶); software used to prepare material for publication: *SHELXL97*.

## Supplementary Material

Crystal structure: contains datablock(s) global, I. DOI: 10.1107/S1600536814005650/hb7209sup1.cif

Structure factors: contains datablock(s) I. DOI: 10.1107/S1600536814005650/hb7209Isup2.hkl

Supporting information file. DOI: 10.1107/S1600536814005650/hb7209Isup3.cml

CCDC reference: 991427

Additional supporting information:  crystallographic information; 3D view; checkCIF report

## Figures and Tables

**Table 1:** Hydrogen-bond geometry (Å, °)

*D*—H⋯*A*	*D*—H	H⋯*A*	*D*⋯*A*	*D*—H⋯*A*
O1—H1⋯O5^i^	0.84 (1)	1.77 (1)	2.5929 (15)	165 (2)
N2—H2⋯O4^ii^	0.87 (1)	1.88 (1)	2.6693 (15)	151 (2)
C5—H5⋯O3^iii^	0.93	2.56	3.3570 (17)	143
C9—H9⋯O2^iv^	0.93	2.57	3.2009 (18)	126

Symmetry codes: (i) 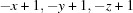; (ii) 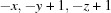; (iii) 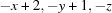; (iv) 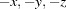.

**Fig. 1.**
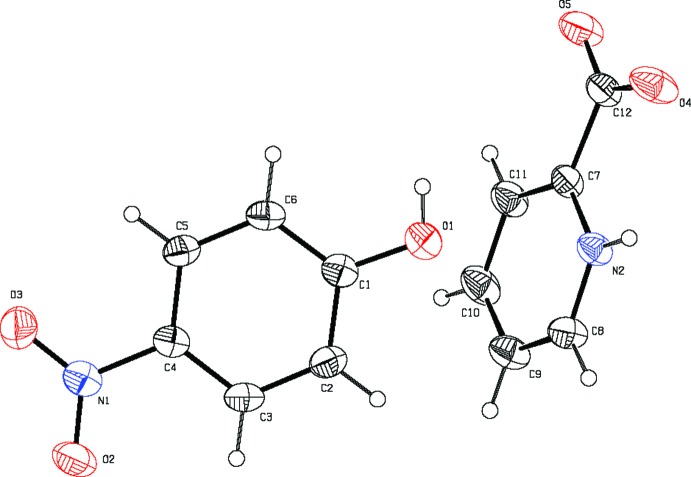
The molecular structure of (I), with 30% probability displacement ellipsoids for non-H atoms.

**Fig. 2.**
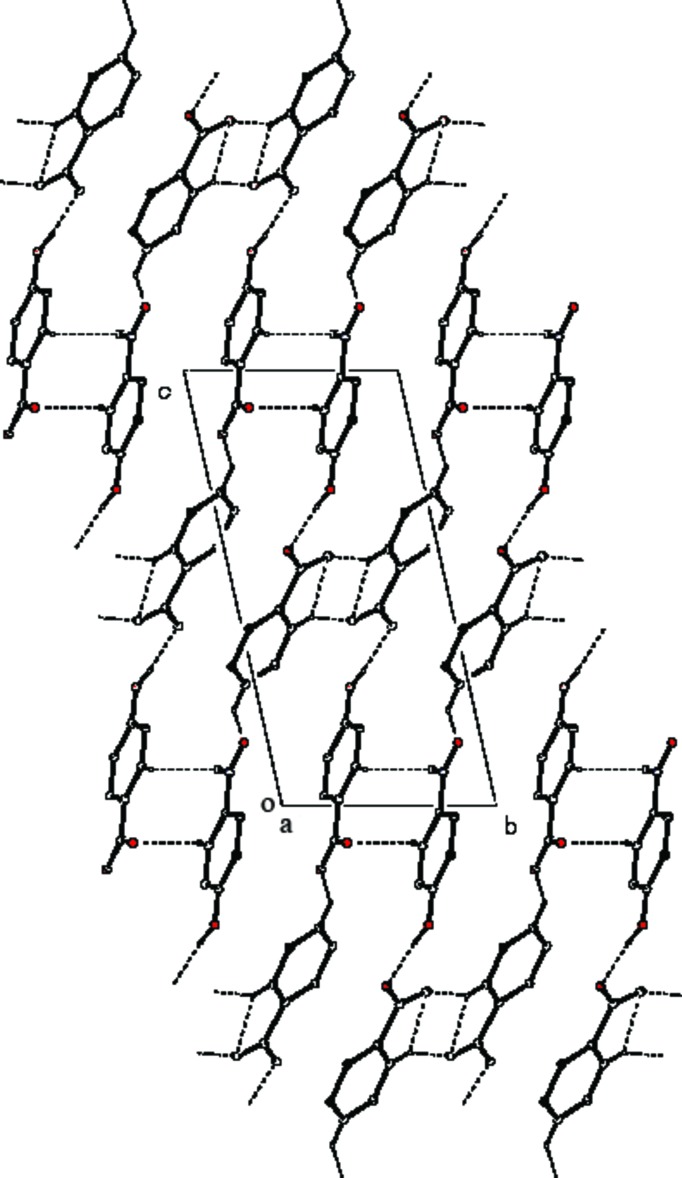
The packing of (I), viewed down a axis. Intermolecular Hydrogen bonds are shown as dashed lines. H atoms not involved in hydrogen bonding have been omitted.

## supplementary crystallographic information

### 1. Comment

We herin report the crystal structure of (I), (Fig. 1). The bond lengths are comparable with those in a similar structure (Pandi *et al.*, 2012).

The pyridine ring is almost planar, with the maximum deviation of 0.005 (2) Å. The carboxy group is twisted at an angle of 2.9 (1)° with the pyridine ring. The nitro group is oriented at an angle of 1.8 (1)° with the benzene ring. The crystal structure features O—H···O, N—H···O and C—H···O (Fig. 2 & Table 1) interactions to form a three dimensional network.

### 2. Experimental

The title material was synthesized by taking 2-carboxypyridine (1.2331 g) and p-nitrophenol (1.3911 g) in an equimolar ratio. 2-carboxypyridine was added gradually in the saturated solution of p-nitrophenol using methanol with continuous stirring for one hour and white precipitate was obtained. Then, the precipitate was dissolved using the same solvent. The prepared solution was allowed for slow evaporation at room temperature to yield colourless blocks after 10 days.

### 3. Refinement

H atoms for C_aromatic_ were positioned geometrically and refined using riding model, with C-H = 0.93 Å and Uiso(H) = 1.2Ueq(C). H atoms bounded to N and O atoms were fixed from the fourier map and refined freely with the distance restraints: 0.82 (1)Å for O—H and 0.86 (1)Å for N—H.

### Crystal data

**Table:**

C_6_H_5_NO_3_·C_6_H_5_NO_2_	*Z* = 2
*M**_r_* = 262.22	*F*(000) = 272
Triclinic, *P*1	*D*_x_ = 1.492 Mg m^−^^3^
Hall symbol: -P 1	Mo *K*α radiation, λ = 0.71073 Å
*a* = 6.1743 (4) Å	Cell parameters from 4728 reflections
*b* = 7.0512 (3) Å	θ = 2.9–27.7°
*c* = 14.2222 (8) Å	µ = 0.12 mm^−^^1^
α = 101.727 (3)°	*T* = 295 K
β = 92.191 (2)°	Block, colourless
γ = 104.758 (4)°	0.32 × 0.24 × 0.20 mm
*V* = 583.60 (6) Å^3^	

### Data collection

**Table:**

Bruker Kappa APEXII CCD diffractometer	3486 independent reflections
Radiation source: fine-focus sealed tube	2327 reflections with *I* > 2σ(*I*)
Graphite monochromator	*R*_int_ = 0.028
ω and φ scan	θ_max_ = 30.4°, θ_min_ = 2.9°
Absorption correction: multi-scan (*SADABS*; Bruker, 2004)	*h* = −8→8
*T*_min_ = 0.963, *T*_max_ = 0.977	*k* = −10→9
13952 measured reflections	*l* = −17→20

### Refinement

**Table:**

Refinement on *F*^2^	Primary atom site location: structure-invariant direct methods
Least-squares matrix: full	Secondary atom site location: difference Fourier map
*R*[*F*^2^ > 2σ(*F*^2^)] = 0.045	Hydrogen site location: inferred from neighbouring sites
*wR*(*F*^2^) = 0.130	H atoms treated by a mixture of independent and constrained refinement
*S* = 1.04	*w* = 1/[σ^2^(*F*_o_^2^) + (0.0602*P*)^2^ + 0.0686*P*] where *P* = (*F*_o_^2^ + 2*F*_c_^2^)/3
3486 reflections	(Δ/σ)_max_ < 0.001
180 parameters	Δρ_max_ = 0.14 e Å^−^^3^
2 restraints	Δρ_min_ = −0.24 e Å^−^^3^

### Special details

**Table:**

Geometry. All esds (except the esd in the dihedral angle between two l.s. planes) are estimated using the full covariance matrix. The cell esds are taken into account individually in the estimation of esds in distances, angles and torsion angles; correlations between esds in cell parameters are only used when they are defined by crystal symmetry. An approximate (isotropic) treatment of cell esds is used for estimating esds involving l.s. planes.

### Fractional atomic coordinates and isotropic or equivalent isotropic displacement parameters (Å^2^)

**Table:**

	*x*	*y*	*z*	*U*_iso_*/*U*_eq_	
C1	0.3865 (2)	0.38442 (18)	0.19001 (9)	0.0451 (3)	
C2	0.2381 (2)	0.2646 (2)	0.11160 (10)	0.0498 (3)	
H2A	0.0853	0.2230	0.1187	0.060*	
C3	0.3152 (2)	0.20783 (19)	0.02422 (9)	0.0474 (3)	
H3	0.2155	0.1288	−0.0281	0.057*	
C4	0.5424 (2)	0.26918 (17)	0.01464 (9)	0.0414 (3)	
C5	0.6929 (2)	0.38640 (18)	0.09160 (9)	0.0459 (3)	
H5	0.8455	0.4274	0.0840	0.055*	
C6	0.6153 (2)	0.44171 (18)	0.17923 (9)	0.0474 (3)	
H6	0.7161	0.5179	0.2317	0.057*	
C7	0.1820 (2)	0.19479 (19)	0.45093 (8)	0.0429 (3)	
C8	−0.1051 (2)	0.1161 (2)	0.32506 (10)	0.0512 (3)	
H8	−0.2284	0.1462	0.2981	0.061*	
C9	−0.0352 (3)	−0.0443 (2)	0.27958 (10)	0.0550 (3)	
H9	−0.1090	−0.1236	0.2211	0.066*	
C10	0.1445 (3)	−0.0868 (2)	0.32101 (10)	0.0589 (4)	
H10	0.1930	−0.1968	0.2912	0.071*	
C11	0.2543 (3)	0.0336 (2)	0.40725 (9)	0.0529 (3)	
H11	0.3772	0.0051	0.4356	0.063*	
C12	0.2879 (2)	0.3378 (2)	0.54544 (9)	0.0477 (3)	
N1	0.6252 (2)	0.21159 (16)	−0.07787 (8)	0.0501 (3)	
N2	0.00405 (19)	0.22997 (16)	0.40848 (7)	0.0452 (3)	
O1	0.29906 (19)	0.44091 (17)	0.27221 (8)	0.0619 (3)	
O2	0.4895 (2)	0.11237 (18)	−0.14600 (7)	0.0689 (3)	
O3	0.82814 (19)	0.26305 (17)	−0.08431 (8)	0.0714 (3)	
O4	0.2047 (2)	0.47818 (17)	0.57395 (7)	0.0701 (3)	
O5	0.44519 (18)	0.29715 (16)	0.58610 (7)	0.0666 (3)	
H2	−0.039 (3)	0.3351 (18)	0.4342 (11)	0.066 (5)*	
H1	0.400 (3)	0.524 (2)	0.3119 (12)	0.085 (6)*	

### Atomic displacement parameters (Å^2^)

**Table:**

	*U*^11^	*U*^22^	*U*^33^	*U*^12^	*U*^13^	*U*^23^
C1	0.0519 (7)	0.0382 (6)	0.0449 (6)	0.0139 (5)	−0.0003 (5)	0.0071 (5)
C2	0.0394 (6)	0.0514 (7)	0.0550 (7)	0.0101 (5)	−0.0036 (5)	0.0075 (6)
C3	0.0460 (7)	0.0433 (6)	0.0476 (7)	0.0104 (5)	−0.0116 (5)	0.0030 (5)
C4	0.0478 (7)	0.0346 (6)	0.0427 (6)	0.0150 (5)	−0.0034 (5)	0.0066 (5)
C5	0.0404 (6)	0.0410 (6)	0.0525 (7)	0.0091 (5)	−0.0041 (5)	0.0054 (5)
C6	0.0476 (7)	0.0409 (6)	0.0469 (6)	0.0078 (5)	−0.0091 (5)	0.0019 (5)
C7	0.0500 (7)	0.0472 (7)	0.0334 (5)	0.0184 (5)	0.0041 (5)	0.0067 (5)
C8	0.0550 (8)	0.0496 (7)	0.0459 (7)	0.0160 (6)	−0.0074 (6)	0.0034 (5)
C9	0.0678 (9)	0.0471 (7)	0.0443 (7)	0.0161 (6)	−0.0051 (6)	−0.0021 (5)
C10	0.0792 (10)	0.0547 (8)	0.0458 (7)	0.0334 (7)	0.0042 (7)	−0.0016 (6)
C11	0.0617 (8)	0.0616 (8)	0.0412 (6)	0.0335 (7)	0.0005 (6)	0.0039 (6)
C12	0.0572 (8)	0.0530 (7)	0.0350 (5)	0.0238 (6)	0.0008 (5)	0.0034 (5)
N1	0.0602 (7)	0.0435 (6)	0.0477 (6)	0.0192 (5)	−0.0009 (5)	0.0071 (5)
N2	0.0544 (6)	0.0435 (6)	0.0386 (5)	0.0206 (5)	−0.0001 (4)	0.0024 (4)
O1	0.0611 (7)	0.0658 (7)	0.0513 (6)	0.0141 (5)	0.0066 (5)	−0.0004 (5)
O2	0.0762 (7)	0.0787 (7)	0.0460 (6)	0.0263 (6)	−0.0106 (5)	−0.0033 (5)
O3	0.0627 (7)	0.0734 (7)	0.0691 (7)	0.0119 (6)	0.0161 (5)	0.0013 (6)
O4	0.0938 (8)	0.0667 (7)	0.0520 (6)	0.0483 (6)	−0.0173 (5)	−0.0117 (5)
O5	0.0763 (7)	0.0785 (7)	0.0471 (5)	0.0448 (6)	−0.0138 (5)	−0.0082 (5)

### Geometric parameters (Å, º)

**Table:**

C1—O1	1.3363 (16)	C8—N2	1.3349 (16)
C1—C6	1.3902 (19)	C8—C9	1.3626 (19)
C1—C2	1.3934 (18)	C8—H8	0.9300
C2—C3	1.3679 (19)	C9—C10	1.364 (2)
C2—H2A	0.9300	C9—H9	0.9300
C3—C4	1.3796 (18)	C10—C11	1.3801 (19)
C3—H3	0.9300	C10—H10	0.9300
C4—C5	1.3813 (17)	C11—H11	0.9300
C4—N1	1.4482 (17)	C12—O4	1.2325 (16)
C5—C6	1.3705 (19)	C12—O5	1.2356 (15)
C5—H5	0.9300	N1—O2	1.2222 (15)
C6—H6	0.9300	N1—O3	1.2252 (15)
C7—N2	1.3352 (16)	N2—H2	0.866 (9)
C7—C11	1.3676 (18)	O1—H1	0.838 (9)
C7—C12	1.5156 (17)		
			
O1—C1—C6	123.25 (12)	N2—C8—H8	120.1
O1—C1—C2	117.49 (12)	C9—C8—H8	120.1
C6—C1—C2	119.25 (12)	C8—C9—C10	119.09 (13)
C3—C2—C1	120.57 (12)	C8—C9—H9	120.5
C3—C2—H2A	119.7	C10—C9—H9	120.5
C1—C2—H2A	119.7	C9—C10—C11	119.88 (13)
C2—C3—C4	119.23 (12)	C9—C10—H10	120.1
C2—C3—H3	120.4	C11—C10—H10	120.1
C4—C3—H3	120.4	C7—C11—C10	119.84 (13)
C3—C4—C5	121.20 (12)	C7—C11—H11	120.1
C3—C4—N1	119.62 (11)	C10—C11—H11	120.1
C5—C4—N1	119.18 (12)	O4—C12—O5	127.49 (12)
C6—C5—C4	119.40 (12)	O4—C12—C7	116.64 (11)
C6—C5—H5	120.3	O5—C12—C7	115.84 (11)
C4—C5—H5	120.3	O2—N1—O3	122.83 (12)
C5—C6—C1	120.31 (12)	O2—N1—C4	118.51 (12)
C5—C6—H6	119.8	O3—N1—C4	118.65 (11)
C1—C6—H6	119.8	C8—N2—C7	123.02 (11)
N2—C7—C11	118.38 (11)	C8—N2—H2	117.9 (11)
N2—C7—C12	116.75 (11)	C7—N2—H2	119.0 (11)
C11—C7—C12	124.87 (12)	C1—O1—H1	109.5 (14)
N2—C8—C9	119.78 (13)		
			
O1—C1—C2—C3	177.46 (13)	C12—C7—C11—C10	179.96 (13)
C6—C1—C2—C3	−1.60 (19)	C9—C10—C11—C7	0.2 (2)
C1—C2—C3—C4	0.5 (2)	N2—C7—C12—O4	−1.96 (19)
C2—C3—C4—C5	0.09 (19)	C11—C7—C12—O4	178.60 (14)
C2—C3—C4—N1	−179.33 (11)	N2—C7—C12—O5	176.51 (12)
C3—C4—C5—C6	0.42 (19)	C11—C7—C12—O5	−2.9 (2)
N1—C4—C5—C6	179.84 (11)	C3—C4—N1—O2	1.36 (18)
C4—C5—C6—C1	−1.53 (19)	C5—C4—N1—O2	−178.07 (11)
O1—C1—C6—C5	−176.89 (12)	C3—C4—N1—O3	−178.10 (12)
C2—C1—C6—C5	2.11 (19)	C5—C4—N1—O3	2.47 (18)
N2—C8—C9—C10	0.7 (2)	C9—C8—N2—C7	0.0 (2)
C8—C9—C10—C11	−0.8 (2)	C11—C7—N2—C8	−0.6 (2)
N2—C7—C11—C10	0.5 (2)	C12—C7—N2—C8	179.91 (12)

### Hydrogen-bond geometry (Å, º)

**Table:**

*D*—H···*A*	*D*—H	H···*A*	*D*···*A*	*D*—H···*A*
O1—H1···O5^i^	0.84 (1)	1.77 (1)	2.5929 (15)	165 (2)
N2—H2···O4^ii^	0.87 (1)	1.88 (1)	2.6693 (15)	151 (2)
C5—H5···O3^iii^	0.93	2.56	3.3570 (17)	143
C9—H9···O2^iv^	0.93	2.57	3.2009 (18)	126

Symmetry codes: (i) −*x*+1, −*y*+1, −*z*+1; (ii) −*x*, −*y*+1, −*z*+1; (iii) −*x*+2, −*y*+1, −*z*; (iv) −*x*, −*y*, −*z*.
